# First step to investigate nature of electronic states and transport in flower-like MoS_2_: Combining experimental studies with computational calculations

**DOI:** 10.1038/srep32690

**Published:** 2016-09-12

**Authors:** Kavita Pandey, Pankaj Yadav, Deobrat Singh, Sanjeev K. Gupta, Yogesh Sonvane, Igor Lukačević, Joondong Kim, Manoj Kumar

**Affiliations:** 1 School of Technology, Pandit Deendayal Petroleum University, Gandhinagar, 382007, India; 2 Inorganic Chemistry Laboratory, University of Oxford, South Parks Road, Oxford OX1 3QR, United Kingdom; 3 Department of Electrical Engineering, Incheon National University, Incheon, 406772, Korea; 4 Advanced Materials Lab, Department of Applied Physics, S. V. National Institute of Technology, Surat, 395007, India; 5 Computational Materials and Nanoscience Group, Department of Physics and Electronics, St. Xavier’s College, Ahmedabad 380009, India; 6 Department of Physics, University J. J. Strossmayer, 31000 Osijek, Croatia

## Abstract

In the present paper, the nature of electronic states and transport properties of nanostructured flower-like molybdenum disulphide grown by hydrothermal route has been studied. The band structure, electronic nature of charge, thermodynamics and the limit of phonon scattering through density functional theory (DFT) has also been studied. The band tail states, dynamics of trap states and transport of carriers was investigated through intensive impedance spectroscopy analysis. The direct fingerprint of density and band tail state is analyzed from the capacitance plot as capacitance reflects the capability of a semiconductor to accept or release the charge carriers with a corresponding change in its Fermi potential levels. A recently introduced infrared photo-carrier radiometry and density functional perturbation theory (DFPT) techniques have been used to determine the temperature dependence of carrier mobility in flower type-MoS_2_. The present study illustrates that a large amount of trapped charges leads to an underestimation of the measured effective mobility and the potential of the material. Thus, a continuous engineering effort is required to improve the quality of fabricated nanostructures for its potential applications.

Recently, along with the rise of research interest in graphene, the search for two-dimensional materials with similar electrical and optical properties has also gained a major attention^1^,^2^,^3^. The family of transition metal dichalcogenides has a layered structure similar to graphene and has shown to be a promising candidate for efficient energy harvesting and storage devices^4^,^5^. Among the various metal dichalcogenides, the semiconductor molybdenum disulphide (MoS_2_) is of great interest because of the ability to fabricate in atomically thin membrane which has a long range of applications^6^,^7^. Being a layered structure with weak van der Walls interaction, it allows the fabrication of layered samples using the chemical exfoliation method or mechanical peeling/cleavage like graphene on insulating substrates^8^. However, in contrast to graphene, MoS_2_ has demonstrated an indirect bandgap of ~1.2 eV for a multilayer structure and a direct bandgap of ~1.8 eV for a single atomic layer structure. The existence of the bandgap has a serious influence on the nature of charge transport and the electronic states^6^,^8^. The electronic, optical, morphological, thermodynamic and vibrational properties along with their applications in catalysis and hydrogen storage have been studied extensively for a few layer MoS_2_ using various approaches and techniques^9^,^10^,^11^,^12^,^13^.

Due to its unique property and application, the synthesis of high purity and large area MoS_2_ nanostructures is always a topic of great interest^14^,^15^,^16^,^17^. Different synthesis techniques like electrospinning, magnetron sputtering, microwave radiation, laser ablation, chemical solution routes and hydrothermal method have been used for the synthesis of MoS_2_^15^,^16^,^17^. The advent of mass production technologies has enabled the scalable growth of MoS_2_, hence showing a commercially low cost viable path for MoS_2_.

However, the reported literature have shown that the room temperature mobility of single and multilayer MoS_2_ is much lower than that of graphene which has been attributed to the bandgap, charge traps and phonon scattering in MoS_2_^1^,^6^,^8^. Understanding of band structure, electronic nature of charge trap and limit of phonon scattering at high temperature will provide a way to improve the mobility or even enhance the mobility to take the full advantage of technological potential of this material^8^,^18^. Here, we present a facile strategy to synthesize a flower-like MoS_2_ nanostructure by one pot hydrothermal method. The present study is also focused on the band tail states, dynamics of trap states and transport of carriers through systematic analysis of impedance spectroscopy and from first principles studies using density functional theory. The complementary modeling and first principles studies allows drawing an insight into device quality such as bandgap and its trap states, and electron-phonon interaction.

## Experimental Section

### Synthesis and characterization of MoS_2_ nanostructures

The flower-like MoS_2_ nanostructures were successfully synthesized using a hydrothermal route. Ammonium molybdate of 0.23 g and thiourea of 0.3 g was dissolved in 20 ml deionized water. The chemicals used in this work i.e. Ammonium molybdate and thiourea are of research grade. The prepared solution was kept under rigorous stirring for 2 hours. The obtained solution was then transferred to a Teflon lined stainless steel autoclave at 220 °C for 22 hrs. The system was then allowed to cool naturally and the black precipitates of MoS_2_ were collected by filtration. The reaction product was washed and centrifuged with distilled water and ethanol to ensure the removal of residual reactants followed by vacuum drying in oven for 24 hrs at 60 °C. The obtained MoS_2_ powder was then dried at 60 °C for 12 hours.

The structural analysis of the obtained MoS_2_ was done by using Panalytical X-Ray diffractometer with CuK_α_ radiation (1.5418 A°). The morphology of MoS_2_ was examined using Carl Zeiss field emission scanning electron microscope (FESEM) at an acceleration voltage of 5 kV. The electrochemical properties were evaluated using a pressed pellet of the as-synthesized MoS_2_ powder with a diameter and width of 10 mm and 2 mm, respectively. The impedance measurements were studied at different applied potentials by applying an AC perturbation voltage of 10 mV amplitude in the frequency range of 10 mHz to 100 kHz.

The first principles calculations based on Density Functional Theory (DFT) as implemented in the Vienna ab-initio simulation package (VASP) were used for computational studies. The exchange-correlation interaction was treated within the generalized gradient approximation (GGA) with the Perdew-Burke-Ernzerhof (PBE) and local density approximation (LDA) functionals. Projected augmented wave (PAW) potential was employed to describe the electron-ion potential. The kinetic energy cutoff ћ^2^|k + G|^2^/2 m for the plane wave basic set was chosen to be 400 eV. In the self-consistent field (SCF) potential and total energy calculations a set of 20 × 20 × 20 k-points was used for Brillouin Zone (BZ) integration in the k-space. The total convergence criteria for SCF iterations were set to be 1 × 10^−6 ^eV and all atomic positions and the unit cell were optimized using the conjugate gradient method until the atomic forces were less than 10^−3 ^eV/Å. To calculate the phonon spectra, density functional perturbation theory (DFPT) implemented in Phonon code was employed. The dynamic matrix was estimated on a 3 × 3 × 3 mesh of q-points in the Brillouin zone.

## Results and Discussion

### Morphological, elemental and structural analysis

**Figure 1:**
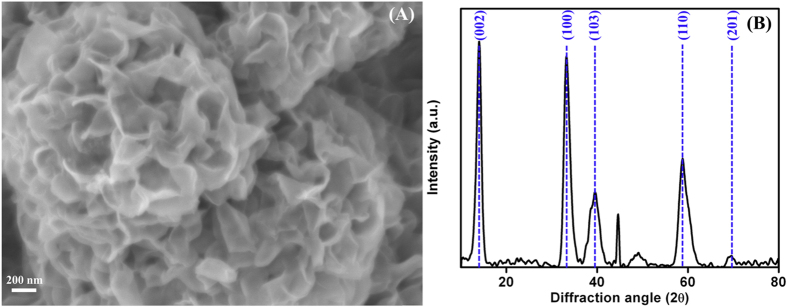
(**A**) High-magnification FESEM image and (**B**) XRD spectrum of MoS_2_ nanostructures.

Figure 1A shows the high magnification FESEM image of interconnected and self-assembled nanosheets of MoS_2_ with various folds. The as-synthesized MoS_2_ powder consists of several individual flower-like spherical shaped particles with an average grain size of ~300 to 350 nm. The average grain size of the nanostructures was estimated from ImageJ software. The possible mechanism for the formation of flower-like MoS_2_ nanostructures is suggested as follows: MoO_4_^−^ and ammonium ions released from the ammonium molybdate and sulphur released from thio urea during the hydrothermal synthesis acts as a source for the formation of MoS_2_ nanostructures. These MoO_4_^−^ ions react with sulphur ions to form MoS_2_ while the interaction of residual ammonia prevents the stacking of MoS_2_ nanostructures^19^,^20^.

The structural properties of the as prepared MoS_2_ nanostructures were studied by XRD measurement. Figure 1B illustrates the XRD pattern of MoS_2_ nanostructure powder in the range of 2θ values from 10°−80°. The XRD spectra of MoS_2_ nanostructure shows the intense diffraction peak at 2θ values of 14.1° and 33.6° which corresponds to the reflections from (002) and (100) plane of hexagonal MoS_2_. Along with the major peaks, the minor peaks at 2θ values of 39.2°, 45.2°, 50.1°, 59.4° and 70.6°corresponds to the reflection plane (103), (104), (105), (110) and (201)^16^. The obtained diffraction pattern is in well agreement with MoS_2_ (JCPDS 37-1492) and reports by other authors^20^,^21^. By using the XRD spectra shown in Fig. 1B and the expression 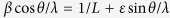, where *β* is the full width at half maxima, *ε* is the lattice strain and *L* is the crystallite size, the average crystallite size of MoS_2_ can be obtained^22^. By taking the reciprocal of intercept on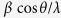 axis the average crystallite size of ~76 nm is obtained in the present study.

**Figure 2:**
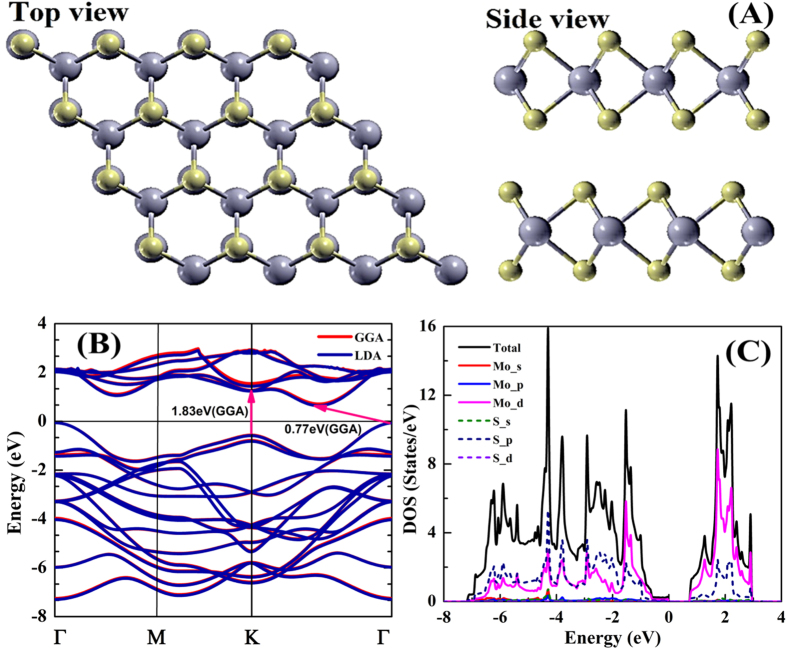
(**A**) The crystal structure of 3D flower type MoS_2_. The gray color indicate Mo-atoms and yellow color indicate S-atoms. (**B**) Electronic band structure and (**C**) Total and partial density of states (DOS) using GGA pseudopotentials.

The crystal structure of 3D flower type MoS_2_ which has the space group P6m1 (D_6h_) is represented in Fig. 2A and forms flower-like 3D shape. The detailed explanation on the difference between flower-like MoS_2_ and well known mono-layer MoS_2_ alongwith the reason that this model corresponds to the studied 3D structures of MoS_2_ is given in the Supplementary Information. In our calculations, we have taken equilibrium lattice constants *a* = *b* = 3.127 Å and *c* = 12.066 Å, which were obtained computationally by relaxing the structure which is in good agreement with Gaur *et al*.^23^. The calculated electronic band structure of 3D flower type MoS_2_ along the high symmetry directions in the first Brillouin Zone (BZ) is shown in Fig. 2B. The minimum energy gaps in 3D flower type MoS_2_ are represented by the arrows. The flower-like MoS_2_ have a conduction band minimum (CBM) between K and Г points and a valence band maximum (VBM) at Г point. The indirect band gap width is 0.77 eV (GGA) and 0.70 eV (LDA). The experimentally obtained energy band gaps for 3D flower type MoS_2_ are in the range of 1.23–1.29 eV^24^,^25^ and the commonly cited theoretical energy band gaps are in the range of 0.70–1.15 eV in agreement with the present study^22^,^26^,^27^,^28^. We have also calculated total density of states (TDOS) and partial density of states (PDOS) shown in Fig. 2C, which defines the nature of the band structure. In the valence band region, between −7.00 eV and −2.10 eV, hybridized S-3p/Mo-4d states are dominant. In the band from −2.10 eV to 0.00 eV, Mo-4d states are dominant near the Fermi level. Generally, Mo-4d and S-3p states are showing the major contribution in the total DOS with respect to the other states. The calculated electronic band gap and refractive index with GGA and LDA functional is listed in Table 1. The obtained refractive index of the 3D flower type MoS_2_ in the parallel and perpendicular direction is 4.09 and 3.60, respectively.

### Phonon spectra and Phonon density of states

**Figure 3:**
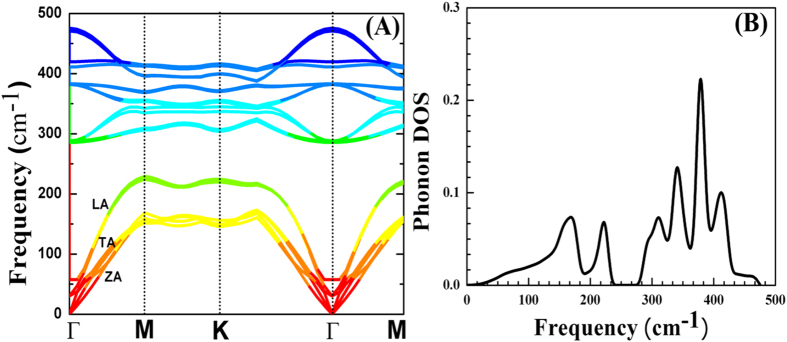
Phonon dispersion and phonon DOS for 3D flower type MoS_2_. The phonon modes of LA, TA and ZA phonon branches also presented.

**Figure 4:**
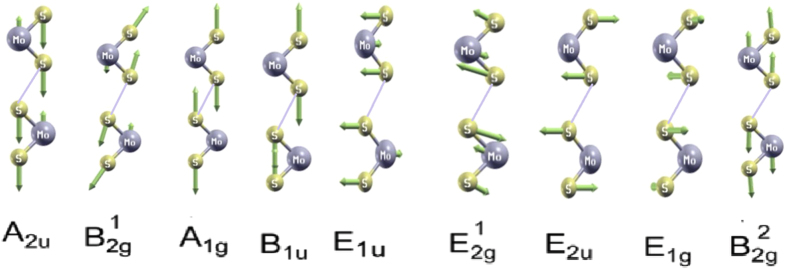
In-plane phonon modes 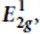 E_2u_, E_1g_ and E_1u_ and the out-of-plane phonon mode A_2u_, 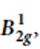 A_1g_, B_1u_ and B_2g_^2^ for the 3D flower type MoS_2_.

Furthermore, phonon spectra and phonon DOS calculations for 3D flower type MoS_2_ nanostructures were also performed. The results of phonon dispersion along the high symmetric points in the Brillouin zone system are shown in Fig. 3. Since the primitive cell of flower-like MoS_2_ contains six atoms the phonon calculations begin with the determination of the equilibrium geometry. From the phonon spectrum, it is possible to compare the stability and structural rigidity of this system. A total 18 phonon modes was found for the flower-like MoS_2_ nanostructures. A factor group analysis of the point group (D_6h_ 3D flower type MoS_2_) shows the long-wavelength optical phonon modes at the Г point (without the three translational acoustic modes). All these frequencies are assigned as Raman (R), infrared (IR), and inactive (IN) modes. Here, modes A_2u_, 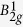, A_1g_, B_1u_, 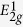, E_1g_ are singly degenerate, while 470.1, 468.3, 409.4, 378.5, 262.8, 253.6 cm^−1^ are their frequencies, respectively and the E_2u_, E_1u_ modes are doubly degenerate with the frequencies 285.7 and 382.4 cm^−1^. The calculated vibrational modes are listed in Fig. 4, and are in good agreement with experimental and theoretical reported values by other authors^29^,^30^,^31^. The R and IR modes are mutually exclusive in flower-like MoS_2_ due to the presence of inversion symmetry in the crystal. The three R active modes in flower-like MoS_2_, A_1g_,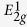, E_1g_, A_2u_, E_1u_ evolve into the IR-active, while the 3D flower type MoS_2_ phonon dispersion has three acoustic modes. Those that vibrate in plane [longitudinal acoustic (LA) and transverse acoustic (TA)] have a linear dispersion and higher energy than the out-of-plane acoustic (ZA) mode (see in Fig. 4). When the wave number *q* increases, the acoustic and low frequency optical branches almost match each other^31^. It is worth mentioning here the absence of degeneracies at the high-symmetry points M and K and the two crossings of the LA and TA branches just before and after the M point. The high-frequency optical modes are separated from the low-frequency modes by a gap of 52 cm^−1^ as shown in Fig. 3 which can also be seen in phonon density of states (DOS)^31^,^32^. There are also three acoustic branches: transverse acoustic (TA), longitudinal acoustic (LA), and out-of-plane acoustic (ZA) branches below the nonpolar transverse optical (TO) and longitudinal optical (LO) modes.

The low-frequency modes B_2g_ and E_2g_ below 60 cm^−1^ (denoted as 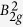 and 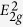 is not shown here and frequency of this mode is 31.0 cm^−1^) in flower-like MoS_2_ have no cousin modes in the monolayer. However, the two modes evolve into a series of shear (S) and compression (C) modes in Few-Layer (FL) MoS_2_ spreading around 30 cm^−1 ^32,33,34,35. These low energy optical phonons are easily activated thermally at room temperature and believed to greatly affect the carrier mobility and thermal conductivity via electron-phonon scattering and phonon-phonon scattering similar as in few layer graphene^35^,^36^.

### Impedance spectroscopy (IS)

**Figure 5:**
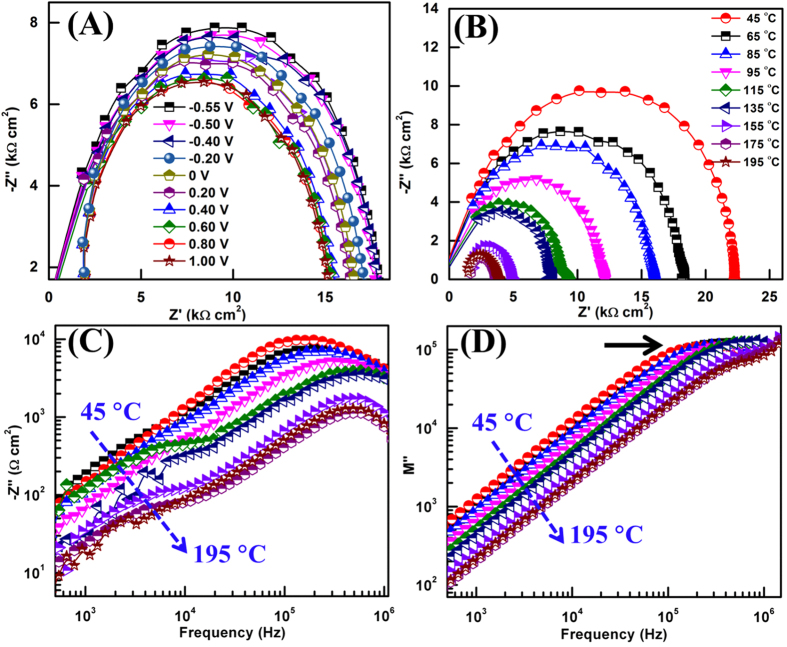
(**A**) Nyquist plots at different applied potential (frequency increases from right to left), (**B**) Nyquist plots at measurement temperatures from 45 °C to 195 °C, (**C**) Temperature dependent imaginary part *Z″* of impedance and (b) imaginary part *M″* of modulus against frequency.

IS spectra was employed to reveal the electronic transport mechanism in flower-like MoS_2_ structure since this technique can provide an information about the nature of electronic states. It is to be noted here that all the IS spectra are recorded under the dark condition in order to avoid the light driven transport in MoS_2_ semiconductor. Figure 5A shows a typical Nyquist spectra recorded at 25 °C for different applied bias. More of these spectra at different temperatures for fixed bias are shown in Fig. 5B. In both the Figures, the symbol and line represents the experimentally measured *Z′* and *Z″* and theoretical fit, respectively. The frequency in these plots increases from the right to left as shown with its corresponding values. In Fig. 5A,B, a single semicircle signifies the presence of single relaxation process in the bulk of material with a relaxation time *τ* (= *RC*).

The other feature of the bias dependent spectra is the shift of relaxation frequency towards the higher frequency region with a decrease in the absolute values of *Z′* and *Z″* as the potential across MoS_2_ nanostructures increases. The Nyquist spectra at low temperature shows a single time constant, however an increase in the temperature causes a distortion in the Nyquist spectra that signifies the involvement of other time constant. In order to deconvolute the presence of different time constants IS spectra are presented in different complex plane formalisms that are interrelated and highlight different aspects of the impedance data^14^,^37^,^38^. An effective way to investigate the properties of MoS_2_ is to combine the spectroscopic plots of imaginary component *Z″* impedance with the electric modulus *M″*. The peaks in *Z″* and *M″* spectroscopic plots can be assigned to the following expression: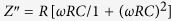 and 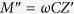 where *C* is the capacitance of empty cell, *ω*  =  2*πf*, *f* is the applied frequency and *Z*′ is the real part of complex impedance^35^. Figure 5C,D shows two time constants in *Z″* vs. frequency plots and the peak shifts systematically towards the lower time scale with the rise in temperature. It is a generalized phenomenon that as the temperature rises, the response of defect states becomes slower and the conduction across the semiconductor increases whereas at lower temperature the hopping phenomenon of charge carriers dominates. The peaks in *Z″* and *M″* appear apart in the frequency spectrum indicating the short range conduction. Moreover, with the increase in temperature the conductivity of semiconductor increases which results in the decrease of the amplitude of *Z″*.

### Density and band tail states

**Figure 6:**
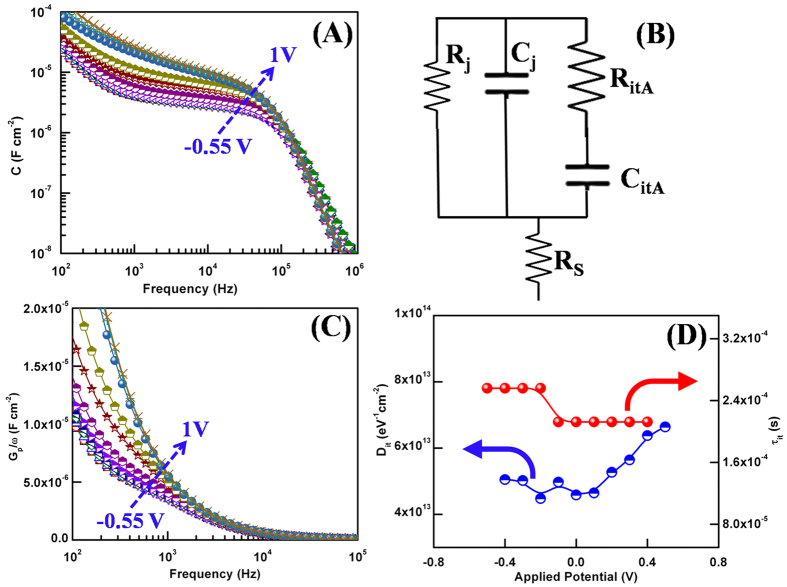
(**A**) Variation of capacitance as a function of frequency at different applied potential, (**B**) The equivalent circuit model of the device, (**C**) Extracted AC conductance over angular frequency *G*_*P*_/*ω* as a function of frequency *f* at various voltages and (**D**) Density and time constant of trap states as a function of voltage. The results are extracted from the capacitance *C* and AC conductance *G*_*P*_.

The direct fingerprint of density and band tail state could be observed in the capacitance plot as capacitance reflects the capability of semiconductor to modulate the charge carriers i.e. accept or release with a change in its Fermi potential levels^6^,^39^. The measured capacitance as a function of frequency and applied potential is shown in Fig. 6A. *C* vs. *f* plot in Fig. 6A presents a rich capacitance pattern but it is not possible to straight forward resolve these different responses. The predominantly observed hump at mid frequency indicates the trap level having a time constant^6^,^14^,^38^. The random distribution of trap charges or structural defects develops an inhomogeneous potential distribution over the semiconductor which leads to a smearing of band gap edges and results in the formation of band gap tail states^6^,^18^. In practice, the inhomogeneity could result from semiconductor itself like sulphur vacancies in MoS_2_^6^,^40^, interfaces and structural defects such as dislocations and grain boundaries.

The *C* vs. *f* plot has been theoretically fitted with the proposed equivalent circuit in Fig. 6B. The fitting parameters at different applied bias are given in the Table S1 of Supplementary Information. In the equivalent circuit, it is considered that the localized state traps has its own time constant and is equivalent to additional series resistance and capacitance in parallel to semiconductors resistance and capacitance. Herein the dominated hump in *C-f* plot is denoted by the level A. The net equivalent device model circuit is shown in Fig. 6B with the net terminal impedance given by,


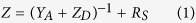


where *Z*_*D*_ is the parasitic impedance of device, *R*_*S*_ is the series resistance due to contacts. 
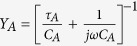
where *C*_*A*_ and *τ*_*A*_ are capacitance and time constants of trap. The trap capacitance is related to the occupancy of electronic states in flower-like MoS_2_ by following a given density of states. The electronic DOS in relation with capacitance is given by
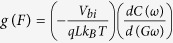
. Here *V*_*bi*_ is the built in voltage due to work function offset and *L* is the semiconductor thickness. The lines in Fig. 6A are the fits using the model showing an excellent match between theoretical and experimental data. From the fitted capacitance plot, the corresponding density of traps and time constant as a function of applied potential has been shown in Fig. 6D.

**Figure 7:**
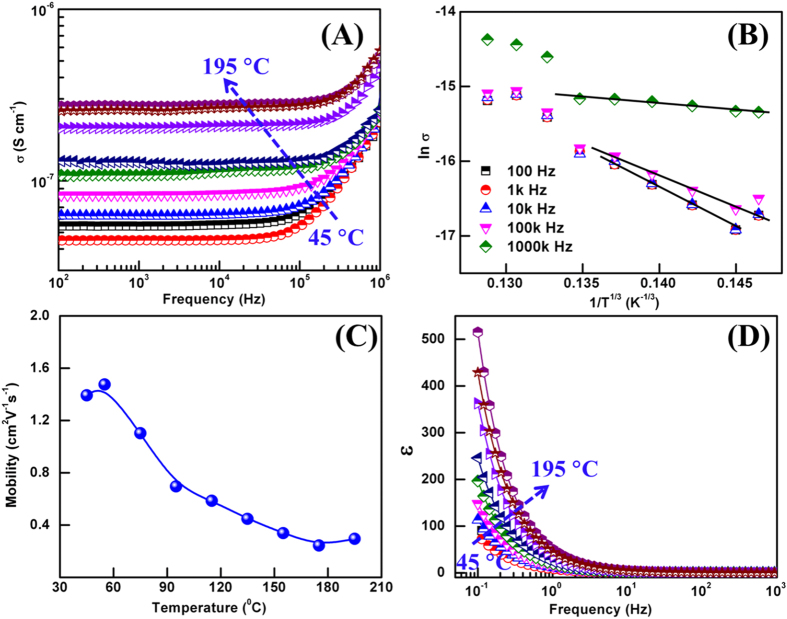
(**A**) Frequency dependent conductivities at different measurement temperatures, (**B**) conductivities of MoS_2_ plotted against temperatures, (**C**) Effective mobility as a function of temperature and (**D**) Variation of dielectric constants with frequency at different measurement temperatures.

To further confirm the obtained values of density of traps and corresponding time constant these values are extracted from AC conductance (*G*_*P*_)^6^. The AC conductance plot over angular frequency (*ω*) as a function of frequency and applied bias is shown in Fig. 7A. The plot of AC conductance depicts a single hump over the probed bias range confirming a single time constant. The mathematical relation between *G*_*P*_ (*ω*) and trap density is given by the following relation^6^,^41^:


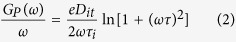


and


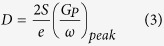


from the above expression, the corresponding time constant is given as 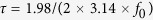. A comparative plot of *D* and *τ* obtained from two different methods in Fig. 6D shows a good agreement between the obtained values.

### Transport Mechanisms

AC conductivity of flower-like MoS_2_ were calculated using the impedance data shown in Fig. 6B and the expression
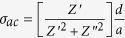
, where *d* is the thickness of pallet and *A* is the cross section area. The frequency dependent variation of *σ*_*ac*_ in the probed temperature range is shown in Fig. 7A. At all the probed temperature, *σ*_*ac*_ follows a frequency independent behavior at low frequency, which is followed by a frequency dependent region at higher frequency. The frequency at which *σ*_*ac*_ slope curves starts to change is the characteristic of the hopping frequency^14^,^38^. The obtained plot of *σ*_*ac*_ follows the Jonschers power law and is given as 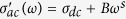 where *σ*_*dc*_ is the dc conductivity that is frequency independent, and *B* and *s* are temperature dependent constants^38^. In literature, the *σ*_*dc*_ values at room temperature for monolayer MoS_2_ is cited in the range of 10^−4 ^Scm^−1^ for single crystal perpendicular to basal plane^14^,^38^, while the *σ*_*dc*_ values for MoS_2_ pallets are commonly reported in the range of 10^−7^ to 10^−6 ^Scm^−1^ 38. In the present study, the *σ*_*dc*_ values are comparable to MoS_2_ pallets but somewhat lower than the single crystal MoS_2_. In the exponential *σ*_*ac*_ region i.e. beyond the hopping frequency, non-linear fitting based on power law was employed to find the variation of *s* with the temperature. It was found that *s* increases as the temperature increases implying that some other conduction mechanism dominates the VRH mechanism.

The dependence of *σ* on temperature at different selected frequency is shown in Fig. 7B and is defined by the expression 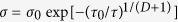 where *σ*_0_ is the pre-exponential factor, *τ*_0_ is the characteristic frequency and is given as 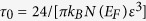 where *k*_*B*_ is the Boltzmann constant, *N*(*E*_*F*_) is the density of localized states at Fermi level and *ε* is the localized length. It can be seen that *σ* of MoS_2_ follows the 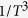 dependency at high temperature region. The localized density of states and states above it are separated from each other by mobility edges in the system. The concept of “mobility edge” has facilitated the understanding of electronic transport in a disordered system^39^,^41^. The studies by Zhu *et al*. and Mott *et al*.^7^,^41^,^42^ have shown experimentally and theoretically that mobility edge depends upon the Fermi level potentials of the semiconductor. During the smearing of the charge carriers, Fermi level lies in the localized region. In our present study, the obtained density of states in Fig. 6 corresponds to the flower-like and not to the surface. A similar conclusion is also drawn by Ahmed *et al*.^13^ for larger number of MoS_2_ flakes. The studies on the MoS_2_ flakes suggest that the nano-flakes higher than 10 in numbers represent a bulk property and Fermi level also shifts towards the bulk.

In general, to define the transport coefficient of semiconductor the drift mobility and effective mobility are commonly used^6^,^8^,^14^,^18^. Understanding this factor influences the mobility and finding a way for mobility engineering to further enhance would allow to explore the technological potential of the material^18^. Figure 7C shows the mobility of flower-like MoS_2_ as a function of potential at different temperatures. The measured mobility is consistent with the results for bulk MoS_2_ devices and is lower than the phonon limited intrinsic mobility in monolayer MoS_2_^14^,^18^. With an increase in temperature, decrease in the mobility of MoS_2_ nanostructures has been observed that can be manifested by the temperature dependence of mobility i.e. *μ* ~ *T*^−*γ*^ where exponent depends upon the domination of photon scattering mechanism^3^,^18^. Generally, the exponent *γ* value defines the phonon scattering as follows: *γ* = 1is for acoustic phonon scattering above Bloch Gruneisen temperature and *γ* > 1 is for optical phonon scattering at high temperature. In the present case, the value of *γ* close to 2.6 is found to be consistent with the scattering of the homo polar mode via zero order deformation potential and previous studies on bulk MoS_2_^3^,^18^. In comparison with the previous report on MoS_2_ monolayer, the exponent is found to be lower than the bulk. The present study indicates that the electron-phonon coupling in 3D flower type and single layer MoS_2_ is different. The present study also supports the conclusion drawn by Kristen *et al*.^3^ on their study of phonon limited mobility in MoS_2_. From the first principles study, they have concluded that transition from an indirect band gap in 3D flower type MoS_2_ to a direct bandgap in monolayer MoS_2_ shifts the bottom of conduction band from the valley along Γ-k path to k, k’ valley which leads to a change in electron-phonon coupling and mobility of MoS_2_ nanostructures. In order to get a further insight into the phonon limited mobility and detail treatments over the phonon collision we adopted the thermodynamics studies of MoS_2_ nanostructures by first principles calculations.

The frequency dependent dielectric permittivity (*ɛ*) drawn at selected temperatures for MoS_2_ nanostructures is shown in Fig. 7D. Dielectric permittivity at all temperatures follows the same trend where it increases from low to high as the frequency tends towards the lower value. The higher value of dielectric constant at low frequency is attributed to the fact that interfaces produce more capacitance due to the increase in net dipoles at the interface and this capacitance offers hindrance to the flow of charge carriers. These dipoles at the interface alter the net polarization of the ionic medium which contributes to dielectric constant^13^. However, less time available for complete polarization at higher frequency leads to the periodic reversal of the applied field. Thus, a negligible charge buildup at the interface results in a constant value of *ɛ* at higher frequency. Different polarizations such as interfacial, Debye oriented, atomic, ionic, and electronic polarizations govern the value of dielectric constant at different frequency range. It has been proved in literature that the polarizations like atomic, ionic, and electronic occurs at a higher frequency, Debye oriented polarization at above 104 Hz while interfacial polarization occurs at lower frequencies. Therefore, interfacial polarization prominent at low frequencies are due to the trapped charges at the interface/grain boundaries because these act as a barrier to the flow of charge carriers contributing to more dielectric properties.

**Figure 8:**
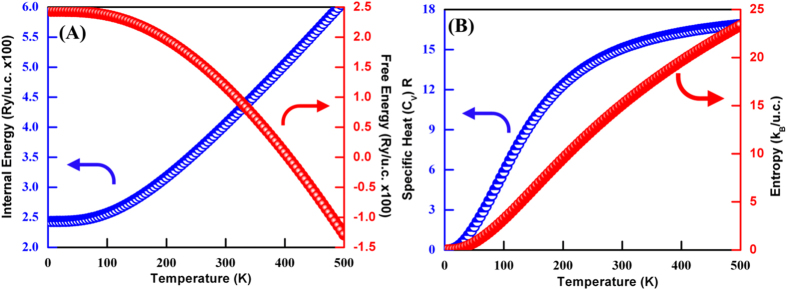
Temperature dependence of internal energy, free energy, entropy, and specific heat at constant volume (*C*_*v*_) of 3D flower type MoS_2_ structure.

The plot for temperature dependence of the internal energy (*E*), free energy (*F*), entropy (*S*), and heat capacity at constant volume (*C*_*v*_) for 3D flower type MoS_2_ is shown in Fig. 8. The temperature variation of thermodynamical functions exhibits, almost, similar trend for the considered MoS_2_ nanostructures. Unfortunately, to the best of our knowledge there is no experimental and theoretical data available for thermodynamic studies of MoS_2_ nanostructures to compare our results with.

The predicted internal energies of 3D flower type MoS_2_ nanostructures as a function of temperature displayed in Fig. 8A suggests that, above 200 K, the internal energies increase almost linearly with temperature and tend towards the *k*_*B*_*T* behavior. Figure 8A shows the free energy versus temperature plot with similar characteristics wherein the free energy decreases gradually with increasing temperature. This behavior is due to the fact that both the internal energy and entropy increases with temperature, which leads to a decrease in the free energy^44^. Also, the inspection of the free energy plots depicts that flower-like MoS_2_ nanostructures are thermodynamically stable in the studied temperature range. The contributions from the lattice vibrations of MoS_2_ to the total specific heat at constant volume (*C*_*v*_) is illustrated in Fig. 8B. From the obtained plot in Fig. 8B, at T < 300 K, *C*_*v*_ increases very rapidly while at T > 300 K, the rate of the change in *C*_*v*_ is somewhat slower and it almost approaches to a constant called Dulong–Petit limit. Moreover, flower-like MoS_2_ nanostructures obey the Debye *T*^*3*^ law at low temperatures. The calculated values of heat capacity at 300 K and 500 K are 15.08 and 16.88 R respectively.

Figure 8B depicts the variations of entropy with the change in temperature for MoS_2_ nanostructures. At 0 K, the entropies of MoS_2_ nanostructures are zero and the change in entropy increases rapidly as temperature increases, while the variation in entropy is small above about 200 K. As entropy of a crystal is caused by an electronic excitation and lattice vibration, the increase in the temperature will lead to the change in entropies^45^. The calculated values of entropy at 300 K and 500 K are 15.11 and 23.34 k_B_/unit cell, respectively. The difference between these values could be responsible for increasing the maximum value of the phonon frequencies of MoS_2_ nanostructures (see Fig. 4). These differences may also stem from the higher contribution of the low phonon frequency^46^. The low-frequency modes, which have longer wavelengths, are associated with larger volumes in the configurational space. Therefore, they cause higher values for specific heat and entropy^45^. In general, it is worth stating that the entropy decreases when the size of the cation mass increases^46^,^47^.

**Figure 9:**
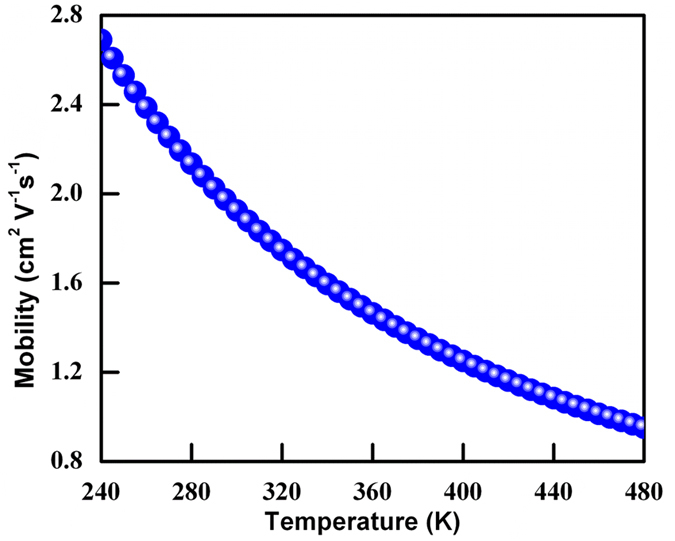
Mobility as a function of Temperature of 3D flower type MoS_2._

Figure 9 shows the theoretically calculated temperate dependence of mobility (*μ*) for MoS_2_ nanostructures. The obtained theoretical data shown in Fig. 9 and obtained practical data in Fig. 7C follows the same trend with the approximate same values of *μ*. The obtained trend and approximately the same values of *μ* signify the reliability of the theoretical modeling. As temperature increases, thermal vibrations (phonons) within a semiconductor increases and causes an increased scattering that leads to a decrease in the carrier mobility^48^,^49^. The temperature dependence of carrier mobility (*μ*) is predicted by the deformation potential theory^49^. However, as mentioned above, experimentally measured dependencies differ from this value of ~T^−3/2^. Reasons for this discrepancy includes: (a) contributions from other scattering mechanisms may be present (for example, at temperatures above 100 K, the contribution of optical phonon scattering becomes considerable, which lowers the value of the mobility); and (b) the non parabolicity, the distortion of equi-energy surfaces as well as the effect of split-off sub-band holes may also contribute.

## Conclusions

A facile strategy to synthesize a flower-like MoS_2_ nanostructure by one pot hydrothermal method is demonstrated. The present study reveals about the band tail states, dynamics of trap states and transport of carriers through systematic impedance spectroscopy analysis and from first principle studies using density functional theory. The calculated electronic band structure, total and partial density of states and optical properties of 3D flower type MoS_2_ were calculated using the first-principles plane-wave pseudopotential method with random phase approximation, based on density function theory. The results show that 3D flower type MoS_2_ is an indirect band gap semiconductor. The indirect and direct band gap is 0.77 eV and 1.83 eV, respectively. Optical anisotropy is observed for light polarizations parallel and perpendicular to the 3D flower type MoS_2_. From the analysis of impedance spectra it can be concluded that as the temperature rises, the response of defect states becomes slower and the conduction across the semiconductor increases whereas at lower temperature the hopping phenomenon of charge carriers dominates.

## Additional Information

**How to cite this article**: Pandey, K. *et al*. First step to investigate nature of electronic states and transport in flower like MoS_2_: Combining experimental studies with computational calculations. *Sci. Rep*. **6**, 32690; doi: 10.1038/srep32690 (2016).

## Supplementary Material

Supplementary Information

## Figures and Tables

**Table 1:** Comparison of electronic band gap of 3D flower type MoS_2_ using different exchange correlation functional and calculated refractive index.

3D flower type MoS_2_	Energy gap (eV)	Refractive index
GGA	0.77 (indirect), 1.83 (direct)	4.09 n(ω)_x, 3.60 n(ω)_z
LDA	0.70 (indirect), 1.83 (direct)	—
