# Supplementary material for: First step to investigate nature of electronic states and transport in flower-like MoS2: Combining experimental studies with computational calculations
Source: Sci Rep. 2016 Sep 12;6:32690. doi: 10.1038/srep32690 (PMC5018888; doi:10.1038/srep32690)
Supplement: Supplementary Information [file srep32690-s1.pdf]

Supplementary Information

First step to investigate nature of electronic states and transport in flower-like MoS_2: Combining experimental studies with computational calculations

Kavita Pandey[1,5], Pankaj Yadav[2], Deobrat Singh[3], Sanjeev K. Gupta[4,*], Yogesh Sonvane[3], Igor Lukačević[6], Joondong Kim[2], Manoj Kumar[1,*]

[1]School of Technology, Pandit Deendayal Petroleum University, Gandhinagar – 382007, India

[2]Department of Electrical Engineering, Incheon National University, Incheon – 406772, Korea [3]Advaced Materials Lab, Department of Applied Physics, S. V. National Institute of Technology, Surat – 395007, India

[4]Computational Materials and Nanoscience Group, Department of Physics and Electronics, St. Xavier's College, Ahmedabad 380009, India

[5]Inorganic Chemistry Laboratory, University of Oxford, South Parks Road, Oxford OX1 3QR, United Kingdom

[6]Department of Physics, University J. J. Strossmayer, 31000 Osijek, Croatia

*Corresponding author: sanjeev.gupta@sxca.edu.in (Dr. Sanjeev K. Gupta)

manoj.kspv@gmail.com (Dr. Manoj Kumar)

Monolayer MoS_2 has a single S-Mo-S layer, while bulk MoS_2 has two such layers, where the Mo atoms in one layer are directly placed above the sulphur atoms of the other layer and vice versa. However, in the case of flower-like MoS_2 the atoms are of nano-dimension. Moreover, their ordered organization during the reaction condition leads to the formation of two dimensional (sheet-like structure) and finally 3D hierarchical flower-like structure. Here, we have obtained the bulk structure using two different layers of MoS_2 with 6 atoms/unit cell as shown in the below figure.

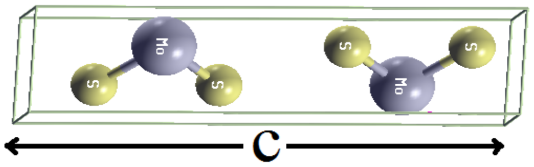

It is clear that within this unit cell both layers of MoS_2 have different atomic arrangements and that continuous repetition of this unit cell generates bulk MoS_2 structure. Importantly, initial structure of the flower-like MoS_2 has been taken from X-ray diffraction crystallographic observation which found reflection peaks from (002) and (100) planes indicating the hexagonal structure of MoS_2. The initial structure was then optimized in an equilibrium condition and the lattice constant of flower like MoS_2 structure was obtained.

Any crystalline object that has height, width and depth in all the three dimension space is called 3D structure while large numbers of atoms combined together is known as bulk structure. Further, from the continuous repetition of the unit cell it is possible to obtain the 3D structure of MoS2. If the unit cell is replicated in all the three directions (x-, y-, z- direction), information regarding the bulk MoS2 can be extracted which is described below:

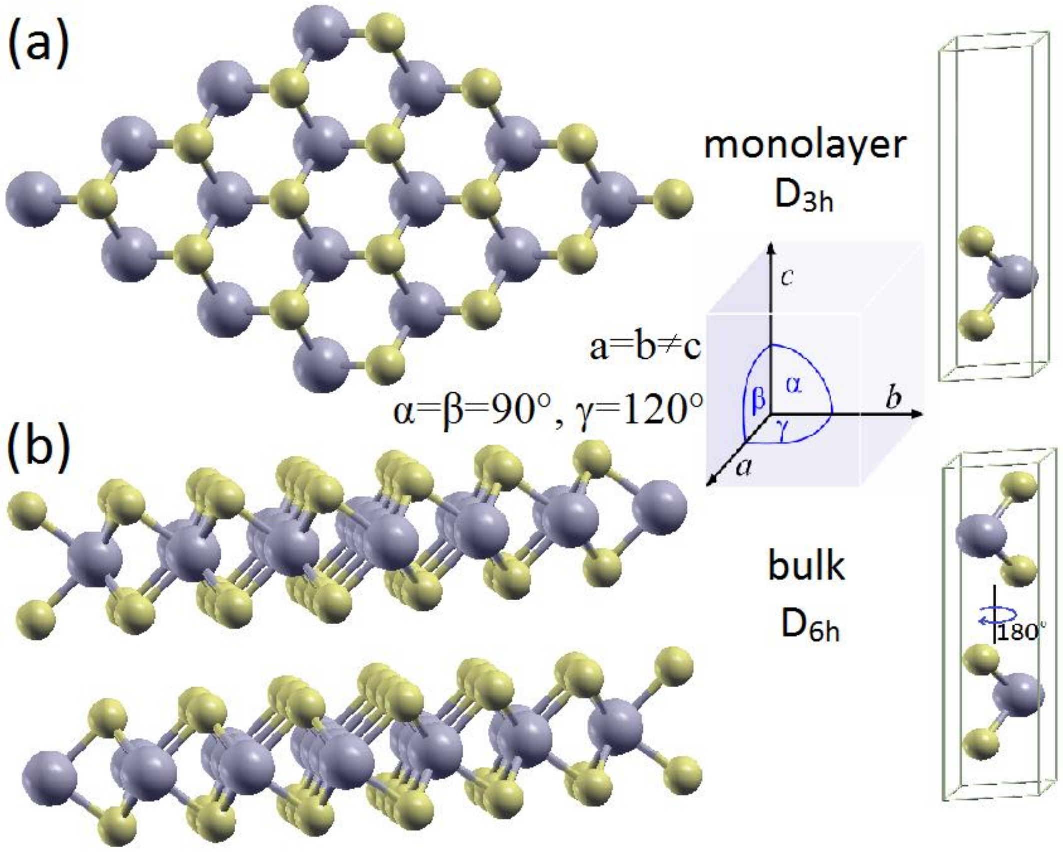

Figure S1. Schematic of the lattice structure of monolayer to bulk transition metal dichalcogenides (TMDs). (a) Top view of monolayer TMD crystal (left) and the unit cell (right), which clearly shows the spatial inversion symmetry breaking in monolayer. (b) Schematic of bulk and even-layer MoS2 structure (left) and the unit cell (right), which has the inversion centre located at the middle plane.

Nevertheless, this spatial inversion symmetry is restored in its bulk form as the weakly van der Waals coupled layers follow the 2H stacking order, in which any two adjacent layers are 180° degree in plane rotation of each other (Fig. S1(b)). The metal atoms of a given layer are sitting exactly on top of the chalcogen atoms of its neighbouring layer, which is well represented by the D6h space group.

Table S1. Various fitting parameters obtained at different potentials by fitting to the equivalent circuit shown in Figure 6B of the main article.

**Applied Potential (V)**	**R_S (kΩ cm^2)**	**R_j (kΩ cm^2)**	**C_j (μF cm^{-2})**	**R_{itB} (kΩ cm^2)**	**C_{itB} (μF cm^{-2})**
-0.55	1.63	24.6	70.1	10.3	196
-0.5	1.60	24.1	69.8	10.0	202
-0.4	1.59	23.4	67.4	9.80	208
-0.2	1.54	20.8	63.6	9.50	216
0	1.53	19.2	59.7	9.42	223
0.2	1.50	18.6	58.1	9.20	233
0.4	1.45	17.4	55.5	9.08	235
0.6	1.43	16.7	52.3	8.85	290
0.8	1.42	15.2	49.9	8.64	300
1	1.36	14.7	45.4	8.36	306
